# Physiological shear flow enhances pinocytosis in human platelets

**DOI:** 10.1038/s41598-026-50030-2

**Published:** 2026-07-02

**Authors:** Masataka Inoue, Kasumi Sagawa, Nobuo Watanabe

**Affiliations:** 1 SIT Research Laboratories, Shibaura Institute of Technology, Saitama, Japan; 2 Biofluid Science and Engineering Laboratory, Department of Bio-Science and Engineering, College of Systems Engineering and Science, Shibaura Institute of Technology, Saitama, Japan

**Keywords:** Platelets, Shear flow, Pinocytosis, Calcium signaling, Flow cytometry, Drug delivery, Biochemistry, Cell biology, Diseases, Medical research, Physiology

## Abstract

Pinocytosis, the uptake of extracellular fluid, is an important yet poorly understood function of platelets. Although platelets are continuously exposed to shear flow in the circulation, the mechanism by which shear modulates pinocytosis in human platelets remains unclear. Here, we investigated the effects of physiological shear rate on platelet pinocytosis using a rotational viscometer combined with flow cytometric quantification using pHrodo dextran. Exposure to 500–1500 s^-1^ shear rates enhanced platelet pinocytosis and increased intracellular Ca^2+^ levels measured using Fluo-4. The pharmacological inhibition of intracellular Ca^2+^ signaling with prostaglandin E1 and chelation of extracellular Ca^2+^ with ethylene glycol tetraacetic acid suppressed both shear-induced Ca^2+^ elevation and pinocytosis, suggesting Ca^2+^ dependence. Notably, shear exposure within this physiological range did not induce classical platelet activation markers, including integrin αIIbβ3 activation or P-selectin expression, suggesting that shear-induced pinocytosis occurs independently of canonical activation pathways. Under plasma-free conditions, platelet pinocytosis was markedly enhanced, even in the absence of shear, without concomitant Ca^2+^ elevation, suggesting the involvement of distinct plasma-dependent regulatory mechanisms. Together, these findings demonstrate that physiological shear promotes calcium-dependent platelet pinocytosis through activation-independent pathways, and highlight shear modulation as a potential strategy for drug loading into platelets in blood cell-based drug delivery systems.

**Supplementary Information:**

The online version contains supplementary material available at 10.1038/s41598-026-50030-2.

## Introduction

Platelets are the blood cells that primarily support hemostasis and enhance hemostasis by interacting with blood proteins and activating coagulation^1^. For example, von Willebrand factor (vWF), fibrinogen, thrombin, and collagen activate platelet αIIbβ3 integrin^2^. Furthermore, as a result of this activation, platelets release adenosine diphosphate (ADP) from their granules along with fibrinogen and tissue factors, thereby further promoting platelet aggregation and blood coagulation^3^. Thus, physiologically active mediators stored in intracellular granules are crucial for platelet hemostatic function.

Pinocytosis, also known as liquid-phase endocytosis, is an important function of platelets. Platelets not only promote hemostasis through secretion, but also uptake plasma proteins via endocytosis^4^. Specifically, they internalize vWF and fibrinogen through the endocytosis pathway and proteins such as albumin through pinocytosis, a nonspecific fluid-phase endocytosis process^5,6^. Paul et al. reported that gelsolin enhances ADP-induced receptor-mediated and fluid-phase endocytosis in platelets^7^. Moreover, they proposed an endocytosis model in which ADP-induced increases in intracellular calcium concentration regulate these enhanced reactions. Such platelet pinocytosis has been associated with drug loading in blood cell-based drug delivery^8,9^. Despite its importance, the regulation of platelet pinocytosis in response to external stimuli remains poorly understood.

The effect of shear flow on pinocytosis in human platelets remains unclear. Platelets are exposed to a physiological range of shear rates within the blood circulation. We have previously reported that 500 and 1500 s^-1^ shear promoted pinocytosis in porcine platelets using FITC-labeled dextran^10^. Nevertheless, several issues remain unresolved in the previous study. Firstly, porcine and human platelets do not consistently exhibit the same reactions. Second, FITC-labeled dextran may adhere to platelet membranes, and the previous flow cytometry results may partially reflect this nonspecific binding. Baxter et al. have also pointed this out, and in their experiments, using pHrodo dextran overcomes this limitation^11^. Third, the pathway by which shear rate enhances pinocytosis remains unclear. Therefore, previous studies did not clarify the effects of shear rate on human platelet pinocytosis and its underlying mechanisms.

To investigate the effects of shear rate on human platelet pinocytosis, we combined quantitative shear rate exposure using a rotational viscometer with a pHrodo-based assessment of platelet pinocytosis. In this study, we further evaluated changes in intracellular calcium concentration using Fluo-4 to examine whether shear rate–dependent modulation of platelet pinocytosis is associated with alterations in intracellular calcium levels. Additionally, calcium suppression was implemented using prostaglandin E1 (PGE1) or ethylene glycol tetraacetic acid (EGTA). The results demonstrated enhanced pinocytosis by human platelets and increased intracellular calcium concentration upon exposure to shear rates of 500, 1000, and 1500 s^-1^. Importantly, under calcium-suppressed conditions, both platelet pinocytosis and intracellular calcium elevation were suppressed, despite shear exposure. In summary, our data suggest a model where exposure to shear rate increases the intracellular calcium concentration in platelets, thereby promoting platelet pinocytosis. These results suggest that platelets may alter the uptake of liquid phase, including proteins, in response to the shear rate within the blood circulation. Furthermore, this study suggests a potential strategy for enhancing drug loading into human platelets by applying shear flow in blood cell-based delivery systems.

## Results

### Shear exposure promotes platelet pinocytosis accompanied by intracellular Ca^2+^ elevation

**Fig. 1:**
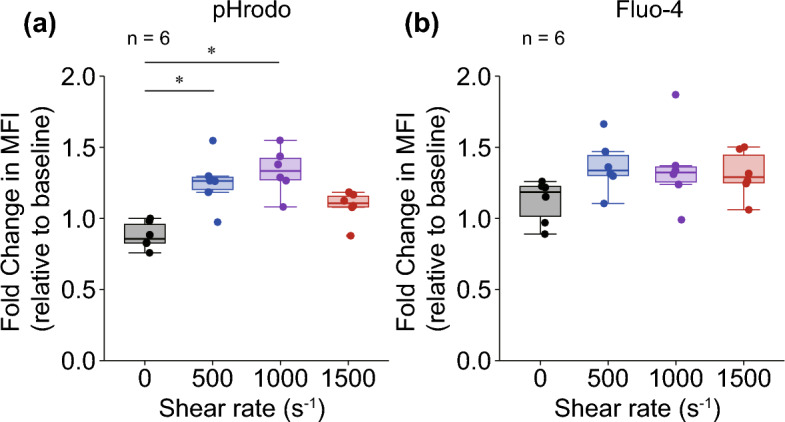
Shear stimulation enhances platelet dextran uptake and intracellular Ca^2^⁺ elevation. Mean fluorescence intensity (MFI) values of (**a**) pHrodo-dextran uptake and (**b**) Fluo-4 were calculated from human platelets (10,000 events) exposed to 0, 500, 1000, and 1500 s⁻^1^ shear, quantified by flow cytometry. Data are expressed as fold-change relative to baseline; platelets incubated in water bath (outside the viscometer) for six donors. Statistical analysis was performed using the Friedman test followed by Dunn–Bonferroni post-hoc comparisons. *p < 0.05. Detailed statistical results are provided in Supplementary Table 1. Full fluorescence intensity histograms of pHrodo-dextran from individual donors are provided in Supplementary Fig. S1.

pHrodo dextran is an established pinocytosis marker whose fluorescence intensity increases upon internalization into cells at a lower pH^11^. In this study, we used pHrodo dextran to investigate whether human platelets enhance pinocytosis in response to shear rate. We collected platelet-rich plasma (PRP) from human volunteers, mixed it with pHrodo dextran, subjected it to shear rates using a rotational viscometer at 37 °C, and measured the fluorescence intensity after shearing. The fluorescence intensity of non-sheared platelets incubated at 37 °C in the water bath served as the baseline. pHrodo uptake by the platelets was evaluated using flow cytometry. We found that exposure to 500, 1000, and 1500 s^-1^ shear enhances pinocytosis in human platelets (Fig. 1(a)). Fluorescence intensity histogram data for all volunteers and the results of the statistical analyses are shown in Supplementary Fig. S1 and Supplementary Table 1, respectively. Although individual histograms displayed modest shifts, quantitative analysis based on MFI revealed consistent increases across donors.

To confirm the intracellular calcium dynamics during shear-induced pinocytosis, we measured calcium fluorescence in platelets pre-incubated with Fluo-4. In the preliminary validation, the calcium fluorescence detected by Fluo-4 disappeared within 5 min of stimulation. Therefore, in this study, calcium fluorescence measurements were performed using flow cytometry immediately after shear exposure. The results are shown in Fig. 1(b) as fold-changes relative to the fluorescence intensity of unstimulated platelets as the baseline, using the mean fluorescence intensity (MFI) of 10,000 cells. The results of the statistical analyses are presented in Supplementary Table 1. Intracellular calcium fluorescence intensity increased under shear conditions (Friedman’s test, p = 0.014). The Dunn–Bonferroni post-hoc test showed no significant difference between the pairs with the lowest values at 500 and 0 s^-1^ (p = 0.096). Although post-hoc comparisons did not remain significant after correction, the data showed a consistent upward trend in Ca^2+^ signals after shear exposure. These results indicate that shear loading is associated with elevated intracellular calcium levels; however, larger sample sizes are required for definitive statistical confirmation. To exclude the possibility that these changes were due to shifts in cell size or density, we analyzed forward scatter (FSC) and side scatter (SSC) MFI values (Supplementary Fig. S2), which showed no significant differences across conditions.

### Shear-induced pinocytosis is calcium-dependent and inhibited by PGE1/EGTA

**Fig. 2:**
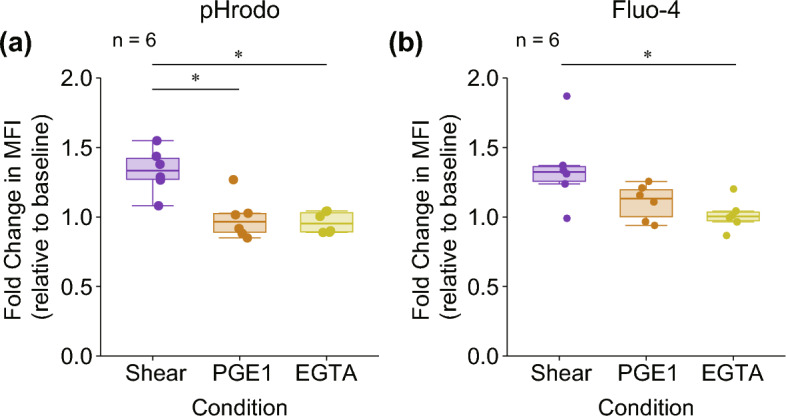
Ca^2^⁺ inhibitors suppress shear-induced platelet dextran uptake and Ca^2^⁺ elevation. Mean fluorescent intensity (MFI) of (**a**) pHrodo-dextran uptake in platelets subjected to 1000 s⁻^1^ shear in the presence of PGE1 or EGTA and (**b**) Fluo-4-based Ca^2^⁺ measurements under identical inhibitory conditions. Data are shown as fold change relative to baseline (n = 6 donors). Statistical analysis: Friedman test followed by Dunn–Bonferroni post-hoc comparisons. *p < 0.05. Exact p-values and effect sizes are listed in Supplementary Table 2. Full histograms of the fluorescence intensities of Fluo-4 in 10,000 events for individual donors are provided in Supplementary Fig. S3.

We examined the dependence of shear-induced pinocytosis in platelets on the intracellular calcium concentration after intracellular calcium suppression with PGE1 and extracellular calcium suppression with EGTA. Calcium inhibition by PGE1 and EGTA strongly suppressed platelet pinocytosis despite exposure to 1000 s^-1^ shear (both p = 0.018, Fig. 2(a); main effects and 95% confidence intervals are shown in Supplementary Table 2). Additionally, PGE1 and EGTA significantly suppressed the increase in intracellular calcium concentration induced by exposure to 1000 s^-1^ shear (Fig. 2(b)). The fluorescence intensity histogram data for Fluo-4 in all volunteers are shown in Supplementary Fig. S3. In summary, calcium inhibition using PGE1 and EGTA reduced shear-induced pinocytosis, suggesting that shear-induced pinocytosis in human platelets is calcium-dependent.

### Shear-induced pinocytosis occurs without classical platelet activation pathways

**Fig. 3:**
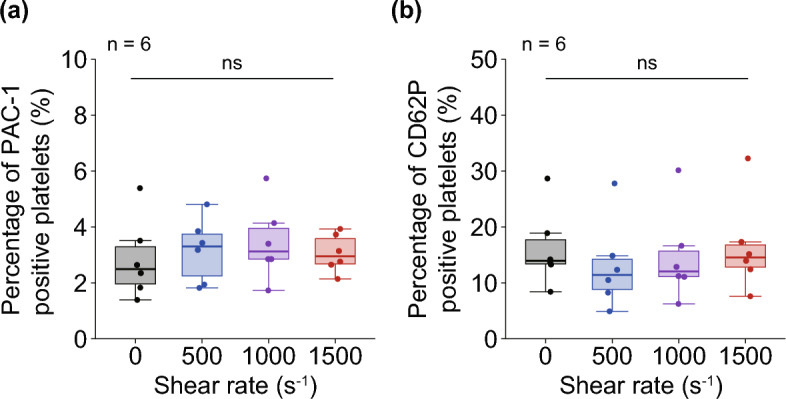
Shear exposure within the physiological range does not induce classical platelet activation markers. Percentage of (**a**) PAC-1-positive and (**b**) CD62P-positive platelets among 10,000 events after exposure to 0–1500 s⁻^1^ shear. Activation thresholds were determined based on adenosine diphosphate stimulation (Supplementary Fig. S4). Data are shown for six donors. Statistical analysis was performed using the Friedman test followed by Dunn–Bonferroni post-hoc comparisons. No statistically significant differences were observed across conditions (ns). Detailed statistical results are provided in Supplementary Table 3. Full histograms of fluorescence intensities of PAC-1 and CD62P in 10,000 events for individual donors are provided in Supplementary Figs. S5 and S6, respectively.

To determine whether shear-induced pinocytosis occurs independently of classical platelet activation pathways, we used an activation-specific anti-integrin αIIbβ3 antibody (PAC-1) and an anti-P-selectin (CD62P) antibody. This analysis was performed to assess whether the observed increase in pinocytosis was accompanied by conventional platelet activation responses. Through preliminary experiments using ADP, we established fluorescence intensity thresholds for platelet activation for each antibody (Supplementary Fig. S4) and the proportion of activated platelets was calculated. Figure 3(a) and (b) show the percentages of activated platelets detected using PAC-1 and CD62P antibody, respectively. The results of the statistical analyses are presented in Supplementary Table 3. Although Friedman’s test suggested an overall difference in CD62P across conditions, post-hoc comparisons for both PAC-1 and CD62P did not reveal significant differences between the individual shear conditions after correction. These results are consistent with the notion that shear-induced pinocytosis is independent of integrin αIIbβ3- and P-selectin-associated classical platelet activation pathways. Fluorescence intensity histograms for PAC-1 and CD62P across all volunteers are shown in Supplementary Figs. S5 and S6, respectively, with no consistent trends observed.

### Plasma removal augments shear-independent pinocytosis without increasing Ca^2+^ levels

**Fig. 4:**
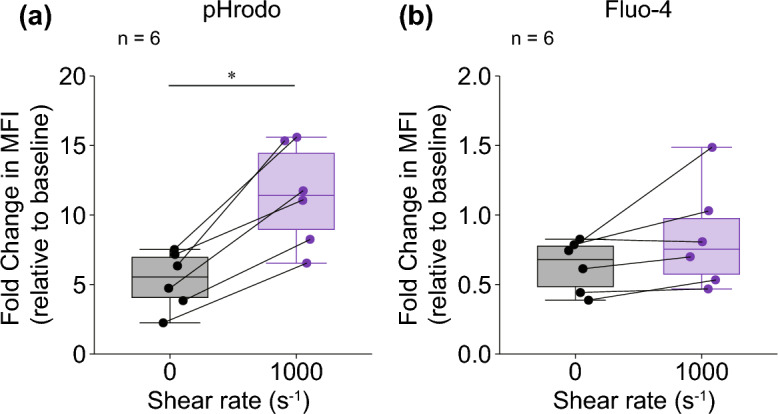
Plasma-free conditions markedly enhance platelet dextran uptake under both static and shear environments. Flow cytometry quantification of (**a**) pHrodo-dextran uptake and (**b**) Fluo-4 signals (MFI) in washed platelets resuspended in HEPES buffer and exposed to 0 or 1000 s^-1^ shear. In plasma-free conditions, dextran uptake was increased under static conditions and was further enhanced by shear, whereas intracellular Ca^2+^ elevation was not detected. Data are shown as fold-change relative to baseline (n = 6 donors). *p < 0.05. Detailed statistical results are provided in Supplementary Table 4.

We performed experiments using washed platelets to evaluate the contribution of plasma components to shear-induced platelet responses,. This approach was designed to distinguish the effects of plasma-derived factors on platelet pinocytosis and intracellular calcium signaling under shear conditions. PRP was centrifuged and washed platelets obtained by replacing the supernatant with N-(2-hydroxyethyl)piperazine-N′-(2-ethanesulfonic acid) (HEPES) buffer were used for comparisons after exposure to 0 and 1000 s^-1^ (Fig. 4). The results of the statistical analyses are presented in Supplementary Table 4. As a result, even under conditions without shear (0 s^-1^), pinocytosis was increased approximately five-fold compared with that in non-sheared platelets incubated under plasma-containing conditions (baseline). Shear exposure at 1000 s^-1^ further increased pinocytosis to nearly ten-fold compared with that in the baseline. On the contrary, intracellular calcium did not increase without (0 s^-1^) or with (1000 s^-1^) shear. This suggests that pinocytosis under plasma-free conditions may be mediated by a calcium-independent mechanism distinct from that observed with PRP. Re-analysis using the 0 s^-1^ condition as the reference did not alter the overall trends or distributions (Supplementary Fig. S7).

## Discussion

Although the importance of platelet pinocytosis has been recognized, the effects of shear exposure on human platelet pinocytosis and its underlying mechanisms remain poorly understood. Here, we demonstrated that shear rates within the physiological range promote human platelet pinocytosis in a calcium-dependent manner. Measurements of platelet activation markers further suggest that this shear-induced pinocytosis is independent of the integrin αIIbβ3- or P-selectin-associated pathways. In addition, experiments performed under plasma-free conditions provide insights into the dual role of plasma components, which appear to enhance shear-induced intracellular calcium elevation and simultaneously suppress platelet pinocytosis. Collectively, these findings support the hypothesis that circulating platelets sense mechanical shear through interactions with plasma components and regulate pinocytosis via calcium signaling. Moreover, this study suggested that shear-enhanced pinocytosis can be exploited for efficient drug loading in platelet-based drug delivery systems.

One of the main findings of this study was that shear exposure enhanced platelet pinocytosis concomitantly with an increased intracellular calcium concentration. Shear-induced pinocytosis has been previously reported in porcine platelets^10^. Consistent with this finding, we confirmed enhanced pinocytosis in human platelets under physiological shear rates accompanied by elevated intracellular calcium levels. Previous studies have also demonstrated calcium mobilization in platelets in response to mechanical stimulation^12^. Mazzucato et al. reported a shear-dependent increase in vWF-mediated platelet calcium concentration^13^. Furthermore, Ilkan et al. observed an increase in intracellular calcium concentration in human platelets under arterial shear rates (approximately 1000 s^-1^) and proposed Piezo1 as a mechanosensitive Ca^2+^ influx channel^14^. Although the precise molecular mechanisms underlying calcium signaling in this study remain unresolved, these reports are consistent with the shear-dependent calcium elevation observed in this study.

The calcium dependence of shear-induced pinocytosis was further suggested using PGE1 and EGTA. PGE1 increases intracellular cyclic AMP levels and inhibits intracellular Ca^2+^ mobilization in platelets^15,16^, whereas EGTA chelates extracellular calcium and suppresses Ca^2+^ influx^13,14^. Accordingly, adding PGE1 and EGTA markedly suppressed shear-induced increases in intracellular calcium even at 1000 s^-1^ shear. Paul et al. proposed an endocytosis model in which elevated intracellular calcium levels promoted platelet pinocytosis via gelsolin-mediated cytoskeletal remodeling^7^. Taken together, these findings suggest that the shear exposure-induced calcium elevation in this study may facilitate pinocytosis through a gelsolin-dependent mechanism. Interestingly, the pharmacological inhibition of calcium signaling may suppress basal pinocytotic activity. Therefore, our data indicate that shear-induced pinocytosis enhancement is calcium-dependent, rather than excluding the broad role of calcium in platelet pinocytosis.

However, in the present study, pharmacological inhibition of calcium signaling using PGE1 and EGTA was examined only under shear conditions (1000 s^-1^). Therefore, additional control experiments under static conditions are required to determine whether these treatments affect dextran uptake independently of shear. In addition, it was not possible to distinguish whether the shear-induced increase in intracellular calcium was due to calcium influx from the extracellular environment or release from intracellular stores. Thus, although our results suggest a role for calcium in shear-induced pinocytosis, the underlying source of calcium remains unresolved.

Despite the shear conditions that promoted pinocytosis (500, 1000, and 1500 s^-1^), integrin αIIbβ3 or P-selectin were not significantly activated. Shear exposure-induced platelet activation has been extensively studied, particularly in the context of mechanical circulatory support, where platelet activation is commonly assessed using PAC-1 and anti-CD62P antibodies^17^. However, most of these studies employed shear stresses exceeding 5 Pa for < 10 s. To the best of our knowledge, no previous study has examined shear-induced platelet activation under physiological shear conditions applied continuously for 30 min. For example, Lu et al. applied 0–20 Pa shear stresses for 120 s and observed significant CD62P and PAC-1 activation at ≥ 15 Pa shear stresses in human platelets anticoagulated with citrate dextrose solution^18^. Although shear exposure in the present study was defined in terms of shear rate, approximate shear stress values were estimated assuming a viscosity of 1.2 mPa·s, yielding stresses of 0.6, 1.2, and 1.8 Pa at shear rates of 500, 1000, and 1500 s^-1^, respectively. Importantly, even when differences in exposure duration and anticoagulant conditions are taken into account, these stress levels remain approximately one order of magnitude lower than those reported to induce platelet activation. Consistently, Chan et al. reported activation of CD42b, a trigger for shear-induced platelet activation, after 15 min of 3 Pa shear exposure^19^. Collectively, these findings support that shear exposure within the physiological range applied in this study was insufficient to induce classical platelet activation.

The dissociation between enhanced pinocytosis and absence of platelet activation suggests that shear-induced pinocytosis may proceed independently of the classical activation pathways. Although activated integrin αIIbβ3 has been implicated in endocytosis^20^, the shear conditions applied in this study promoted pinocytosis without detectable integrin activation. Thus, shear-induced pinocytosis may involve alternative pathways distinct from integrin αIIbβ3-mediated endocytosis. It should also be noted that previous studies have reported shear-induced integrin αIIbβ3 on platelets shedding^21–23^, which may be related to the prolonged shear exposure employed in this study. Notably, despite a tendency toward increased intracellular calcium levels, integrin αIIbβ3 or P-selectin were not activated. These findings suggest that pinocytosis and membrane antigen expression have different calcium thresholds and pinocytosis is triggered by lower calcium levels than those required for platelet activation.

We used washed platelets to isolate the contribution of plasma components to shear-induced platelet responses. Plasma removal markedly enhanced platelet pinocytosis even in the absence of shear flow, without a concomitant increase in intracellular calcium levels. This finding suggests that the regulation of pinocytosis under plasma-free conditions differs fundamentally from the calcium-dependent mechanism observed in PRP. One possible explanation is the presence of plasma-derived factors that suppress platelet pinocytosis under physiological conditions, although such factors have not yet been clearly identified. Another possibility is that these results may indicate that plasma proteins do not inhibit pinocytosis itself, but rather inhibit contact between platelets and dextran. Alternatively, the washing procedure itself may have partially generated procoagulant platelets with phosphatidylserine exposure, which are known to exhibit enhanced dynamin-dependent pinocytosis^11^. Under this scenario, shear-enhanced pinocytosis in plasma-free conditions may occur via a calcium-independent pathway. However, differences in viscosity between PRP and washed platelet suspensions should be considered when interpreting these comparisons. At present, these possibilities cannot be distinguished based on the available data. The PRP samples were anticoagulated with citrate, and the washed platelets were prepared in the HEPES buffer containing 2 mM CaCl₂. Consequently, the results should be interpreted with caution, as differences in extracellular calcium concentrations may influence Ca^2+^ elevation induced by shear stress and the subsequent pinocytosis response.

This study has several limitations. The present study was conducted with a limited sample size of six participants. Consequently, the observed trends should be interpreted with caution, and a larger cohort is necessary to confirm the statistical robustness of these findings. In addition, imaging-based analyses were not performed in this study and would provide direct evidence of dextran internalization at the single-cell level, as well as enable assessment of the temporal and concentration-dependent dynamics of intracellular calcium changes. Such approaches would further strengthen the interpretation of the present findings. Furthermore, additional experiments using calcium-modulating approaches would be beneficial to confirm the calcium-independent mechanism of pinocytosis in washed platelets. A shear loading system using a rotational viscometer does not fully recapitulate the complex hemodynamic environment of blood vessels, including pulsatile flow, spatial shear gradients, interactions with the vessel wall and endothelium, or red blood cell influence. Moreover, sustained constant shear exposure for 30 min did not occur in vivo, and future studies examining platelet pinocytosis in response to transient or pulsatile shear may provide additional insights. Finally, this study should be interpreted as an exploratory analysis providing initial evidence based on a limited dataset. Although we demonstrated shear-associated, calcium-dependent enhancement of platelet pinocytosis, the overall amount of data remains limited and was not validated using complementary approaches such as imaging-based confirmation of dextran internalization or analysis of calcium-dependent signaling pathways. Therefore, the specific molecular mechanisms underlying these observations, particularly with respect to plasma protein interactions, remain to be elucidated. Despite these limitations, our findings provide new insights into shear-induced pinocytosis in human platelets and its relationship with calcium dynamics, and suggest potential applications in platelet-based drug delivery. Future studies with larger sample sizes, multimodal validation (e.g., imaging and signaling analyses), and microfluidic systems that more closely mimic in vivo conditions will be essential to further substantiate and extend these findings.

## Methods

### Reagents

The pHrodo red-labeled dextran (10 kDa) reagent was purchased from Thermo Fisher Scientific (Cat. P10361), Fluo-4 was purchased from DOJINDO LABORATORIES (F311, Fluo 4-AM), anti-human PAC-1 was purchased from Becton Dickinson (Cat. 340,507), and anti-human CD62P (P-selectin) antibody was purchased from BioLegend (Cat. 304,910). PGE1 was obtained from Cayman Chemical Company (Cat. no.745–65-3) and EGTA from DOJINDO LABORATORIES (Cat. 346–01,312). Apyrase (Cat. A7646) and ADP (Cat. 01,905) were purchased from Sigma-Aldrich.

### Human platelet preparation

Blood samples were collected from healthy volunteers using 22-gauge needles into 3.2% sodium citrate. Citrated platelet rich plasma was obtained by centrifugation at 150 × *g* (without brake) for 12 min, referencing prior research^24^ in order to prevent the loss of plasma proteins and platelet reactivity. Fluo-4 AM (5 µM: final concentration) was mixed into the PRP, and the fluorescent dye for calcium signaling was internalized into the cells by incubating at 37 °C for 30 min. All volunteers provided informed consent in accordance with the Declaration of Helsinki, and all experiments adhered to the ethical standards set forth by the Ethics Committee of the Shibaura Institute of Technology (#23–049).

### Shear exposure of platelets

A rotational viscometer (DV2T, Brookfield Corporation, Canada) with a cone-plate geometry was used to generate different shear rates under temperature controlling (37 °C) on platelets. Citrated platelet samples mixed with pHrodo dextran (30 µg/mL: final concentration) were exposed to a physiological range of shear rates (0, 500, 1000, and 1500 s^-1^) for 30 min. An external baseline was defined as non-sheared PRP incubated in a water bath at 37 °C to minimize potential artefacts associated with sample handling and contact with the viscometer surfaces. This condition was intended to represent a non-device-exposed reference, as platelet responses may be influenced by confinement and surface interactions within the viscometer even under nominally static (0 s^-1^) conditions. The order of each shear load was randomized since the resting platelets may change over time during blood storage. Platelet samples removed from the rotational viscometer were immediately subjected to flow cytometry analysis as the fluorescence signal may decrease over time with pHrodo dextran and Fluo-4. Under calcium-inhibiting conditions, PGE1 (final concentration: 100 nM) or EGTA (final concentration: 1 mM) was added to the platelet samples prior to shear exposure.

### Flow cytometry analysis

Platelet samples subjected to shear exposure were diluted 150-fold using calcium- and magnesium-free Dulbecco’s phosphate-buffered saline (PBS: 049–29,793; FUJIFILM Wako Pure Chemical Corporation, Japan) to minimize fluorescence artifacts caused by membrane-bound dextran on the platelets. The fluorescence intensities of pHrodo dextran and Fluo-4 in the platelets were measured using a flow cytometer (FACSAria III; Becton, Dickinson and Company, USA). Single platelets were gated based on the forward scatter intensity to avoid overdetection associated with the fluorescence intensity from doublets. MFI values were calculated from 10,000 gated platelet events for each sample for quantitative analysis. Data were normalized to the baseline condition and expressed as fold change for each donor. In a preliminary study, the time course of pHrodo uptake by baseline platelets was confirmed for 90 min (Supplementary Fig. S8(a)). PRP containing pHrodo was collected at each time point and analyzed immediately after PBS dilution. Because pHrodo uptake reached approximately half of the maximal level at 30 min, we selected 30 min as the standard exposure duration to detect shear-dependent changes in pinocytosis. The time-dependent decay in calcium fluorescence measured using Fluo-4 was confirmed with and without adding ADP (10 µM: final concentration; Supplementary Fig. S8(b)). In this experiment, ADP was added immediately before measurement, and fluorescence was recorded within the indicated time window after stimulation. Because Fluo-4 fluorescence decreased within 5 min after ADP stimulation, intracellular Ca^2+^ signals after shear exposure were measured within 1 min.

To evaluate platelet activation, a separate experiment was conducted on platelets without Fluo-4 and assessed using PAC-1 and CD62P antibodies after shear exposure. Then, 10 µL PAC-1 and 10 µL CD62P antibody were added to platelet samples after shear exposure and antibody binding occurred for 15 min in a water bath at 37 °C. The number of platelets exhibiting a fluorescence intensity above the threshold for PAC-1 and anti-CD62P antibodies was counted and quantified as the percentage of activated cells. The activation thresholds for PAC-1 and CD62P were determined based on measurements of fluorescence intensity in platelets with and without 10 µM ADP addition (see Supplementary Fig. S4).

### Washed platelet preparation (plasma-free conditions)

To examine pinocytosis and calcium responses of platelets in the absence of plasma components, washed platelets were prepared. To inhibit platelet activation caused by the washing process, apyrase (0.1 IU/mL: final concentration) was added to the Fluo-4-treated PRP and centrifuged at 600 × *g* for 5 min. The supernatant of centrifuged PRP was replaced with HEPES-buffered saline (10 mM HEPES (free acid), 135 mM NaCl, 3 mM KCl, 0.34 mM NaH_2_PO_4_, 1 mM MgCl_2_·6H_2_0, pH 7.4, 5 mM D-glucose). Prior to shear loading, pHrodo dextran and calcium chloride solutions (2 mM: final concentrations) were added to the samples. After shear loading, flow cytometry was performed using the aforementioned method. In this study, shear conditions were defined in terms of shear rate, which is determined by the geometry and rotational speed of the viscometer. Therefore, comparable shear rates were applied to both PRP and washed platelet samples, although differences in viscosity between conditions may result in variations in absolute shear stress.

### Statistical analysis

All statistical analyses were performed using the R software (version 4.2.0). Since the sample size was small (n = 6) and normality could not be reliably assessed, non-parametric tests were applied. Wilcoxon signed-rank test was used for paired two-group comparisons. For paired multi-group comparisons, the Friedman test, followed by Dunn’s post-hoc test with Bonferroni correction, was applied. Statistical significance was set at P < 0.05. Effect sizes were reported as rank-biserial correlations and 95% confidence intervals were estimated using bootstrap resampling of paired observations (5,000 iterations).

## Supplementary Information

Supplementary Information.

## Data Availability

All data are presented in this article. The flow cytometry data used in this study, including the gating strategy for platelet activation, are provided in the supplementary file. Other raw data supporting the findings of this study will be made available by the corresponding author upon request.
