# Supplementary material for: Physiological shear flow enhances pinocytosis in human platelets
Source: Sci Rep. 2026 Jul 2;16:20167. doi: 10.1038/s41598-026-50030-2 (PMC13324158; doi:10.1038/s41598-026-50030-2)
Supplement: Supplementary file 1 — Supplementary Information. [file 41598_2026_50030_MOESM1_ESM.pdf]

Supplementary Figures and Tables

Physiological shear flow enhances pinocytosis in human platelets

Masataka Inoue*[1], Kasumi Sagawa[2], Nobuo Watanabe*[2]

[1] SIT Research Laboratories, Shibaura Institute of Technology, Saitama, Japan
[2] Biofluid Science and Engineering Laboratory, Department of Bio-Science and Engineering, College of Systems Engineering and Science, Shibaura Institute of Technology, Saitama, Japan

*Correspondence to: Masataka Inoue (inoue.masataka.w2@shibaura-it.ac.jp), Nobuo Watanabe (nobuo@sic.shibaura-it.ac.jp)

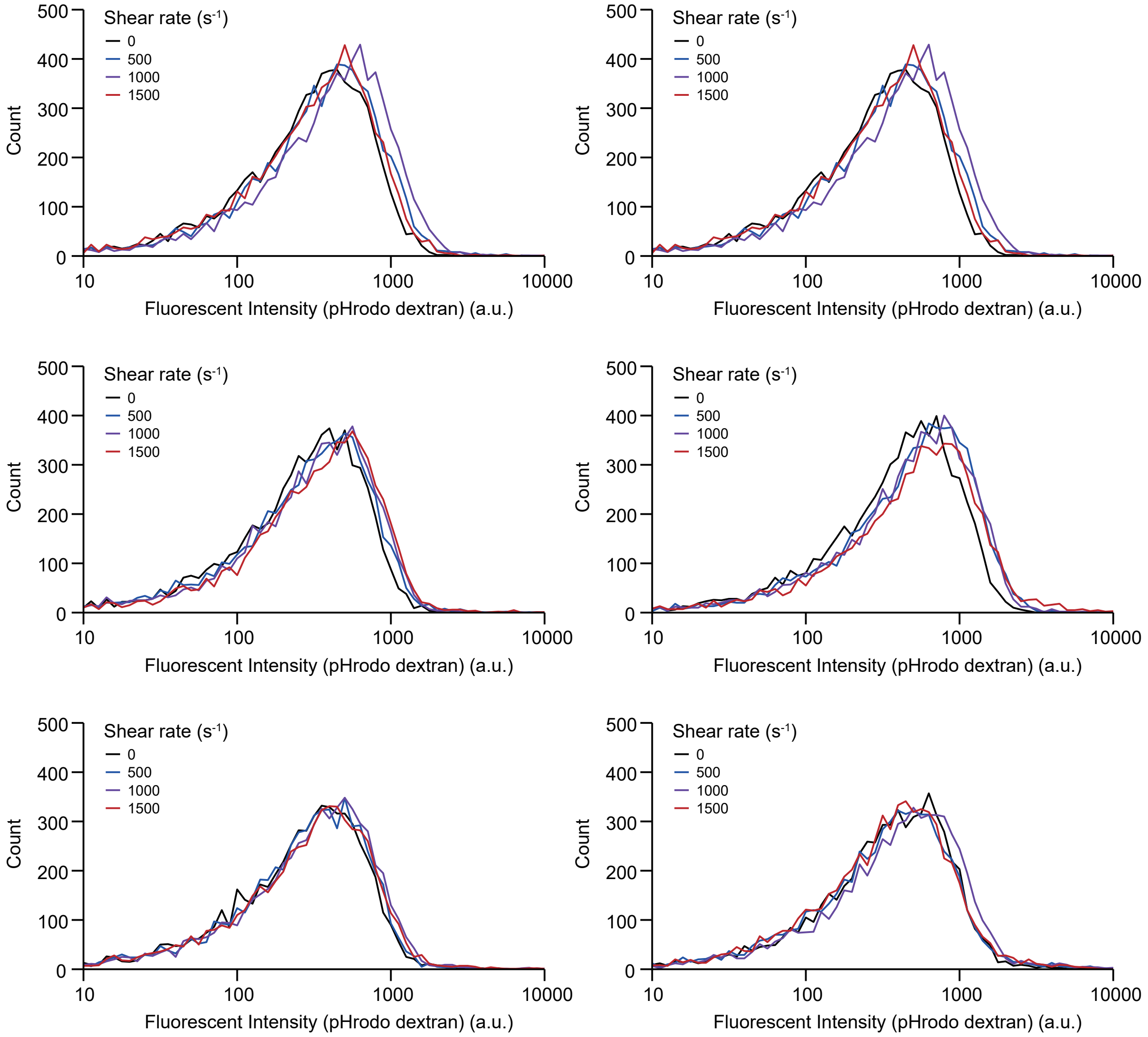
Shear rate (s-1)
0
500
1000
1500
Count
Fluorescent Intensity (pHrodo dextran) (a.u.)

Supplementary Figure S1 | Fluorescence histograms of pHrodo fluorescence under shear for individual donors.

Fluorescent histograms of all six donors (10,000 cells each condition) for pHrodo-dextran at 0, 500, 1000, and 1500 s^{-1}. Corresponding mean fluorescence intensity (MFI) values are summarized in Figure 1(a).

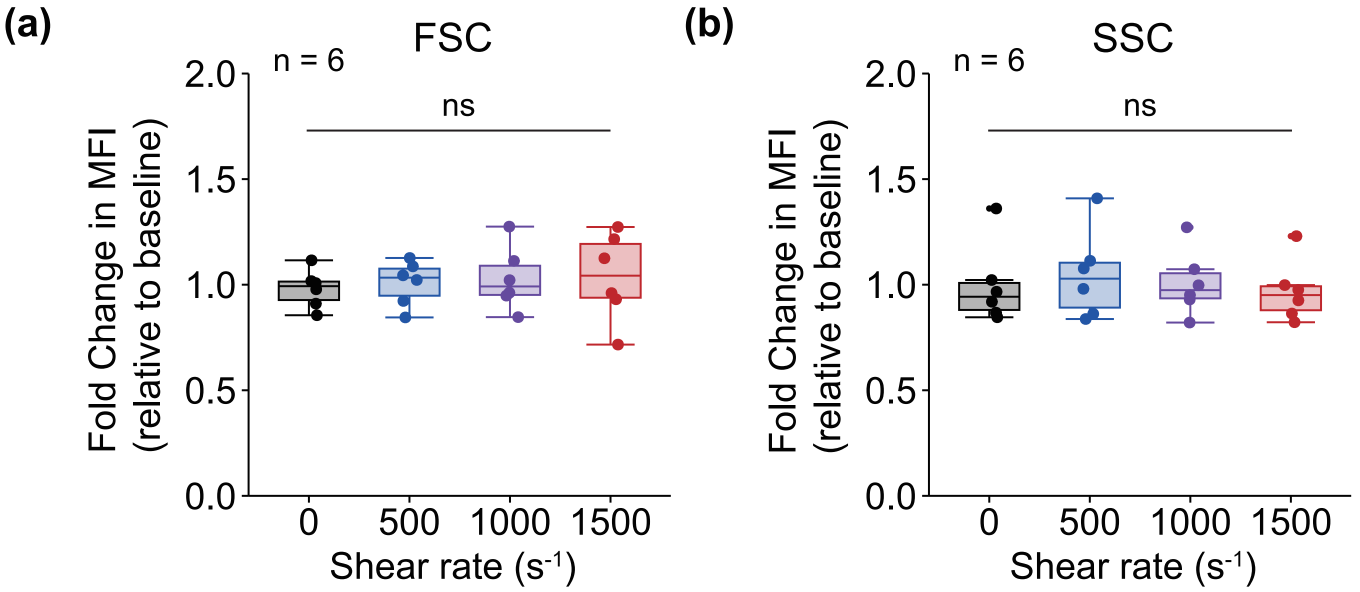
(a)
FSC
n = 6
ns
Fold Change in MFI (relative to baseline)
2.0
1.5
1.0
0.5
0.0
0
500
1000
1500
Shear rate (s^{-1})
(b)
SSC
n = 6
ns
Fold Change in MFI (relative to baseline)
2.0
1.5
1.0
0.5
0.0
0
500
1000
1500
Shear rate (s^{-1})

Supplementary Figure S2 | Shear within the physiological range does not alter forward scatter (FSC) or side scatter (SSC), reflecting stable platelet size and granularity.
The mean fluorescence intensity (MFI) of (a) FSC and (b) SSC was calculated from human platelets (10,000 events) exposed to 0, 500, 1000, and 1500 s^{-1} shear, quantified by flow cytometry. Data are shown as fold change relative to baseline (n = 6 donors). Statistical analysis was performed using the Friedman test followed by Dunn–Bonferroni post-hoc comparisons. No statistically significant differences were observed across conditions (ns).

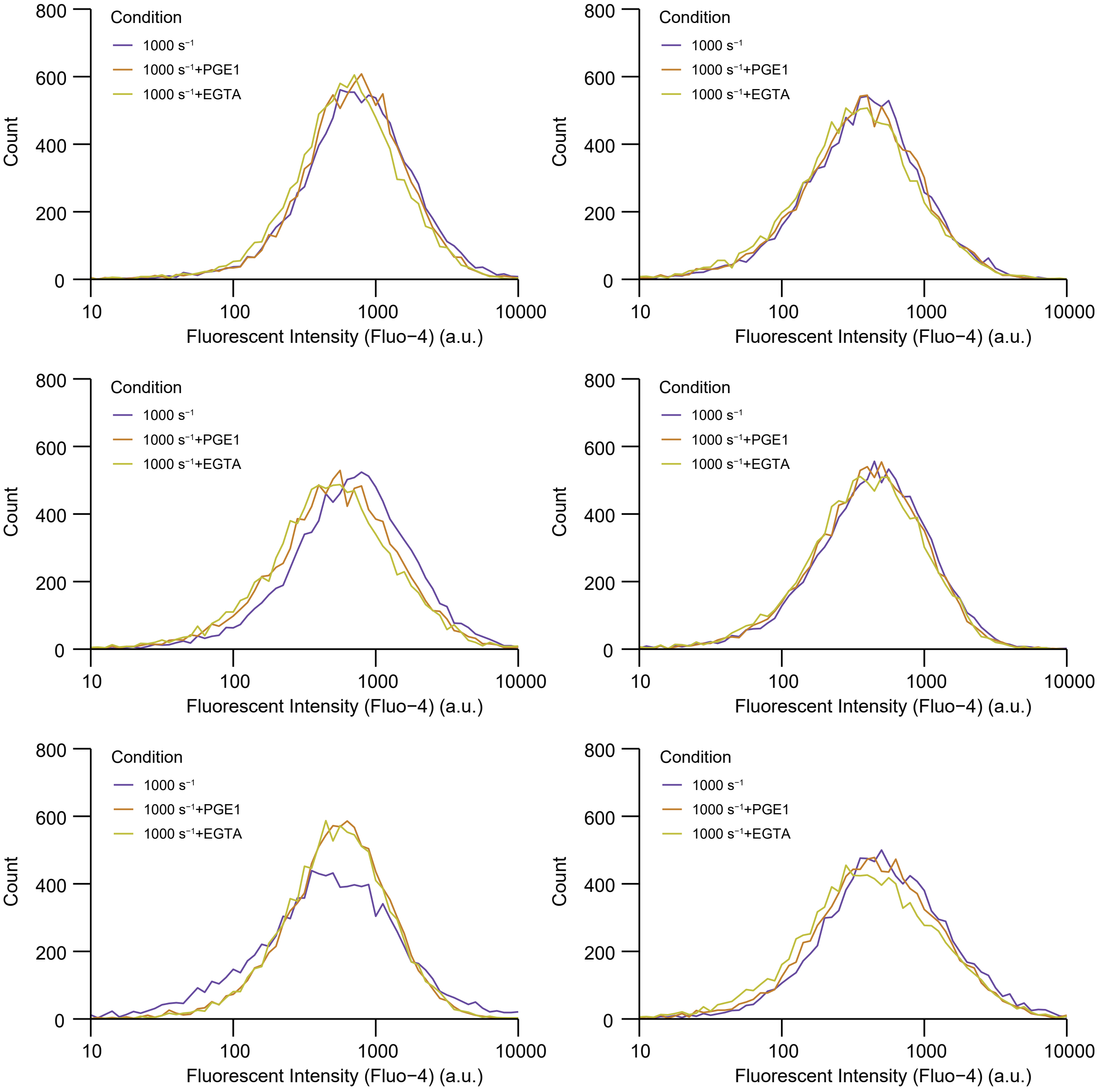
Condition
1000 s⁻¹
1000 s⁻¹+PGE1
1000 s⁻¹+EGTA
Count
Fluorescent Intensity (Fluo−4) (a.u.)
0
200
400
600
800
10
100
1000
10000

Supplementary Figure S3 | Fluorescent histograms of Fluo-4 fluorescence under shear for individual donors.

Fluorescent histograms of all six donors (10,000 cells each condition) for Fluo-4 at 0, 500, 1000, and 1500 s^{-1}. Corresponding mean fluorescence intensity (MFI) values are summarized in Figure 2(b).

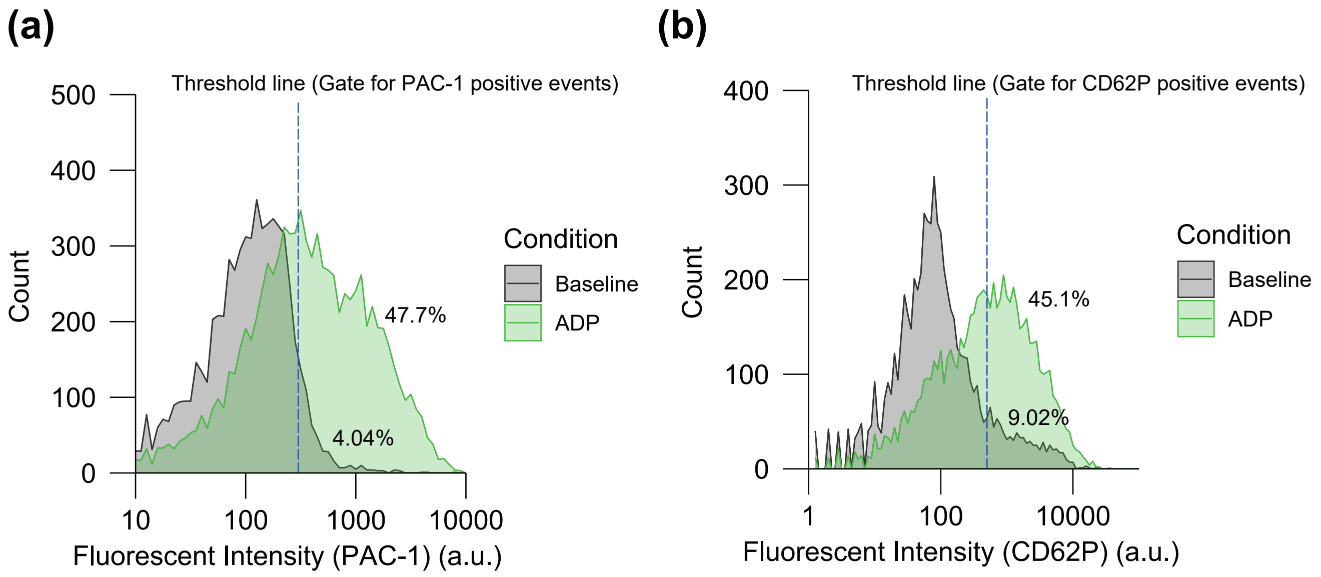
(a)
Threshold line (Gate for PAC-1 positive events)
500
400
300
200
100
0
Count
47.7%
4.04%
10
100
1000
10000
Fluorescent Intensity (PAC-1) (a.u.)
Condition
Baseline
ADP
(b)
Threshold line (Gate for CD62P positive events)
400
300
200
100
0
Count
45.1%
9.02%
1
100
10000
Fluorescent Intensity (CD62P) (a.u.)
Condition
Baseline
ADP

Supplementary Figure S4 | Threshold determination for PAC-1 and CD62P activation in platelets.

Histograms of ADP-stimulated and unstimulated platelets used to define fluorescence thresholds for activation gating. These thresholds were consistently applied in the analyses presented in Figure 3.

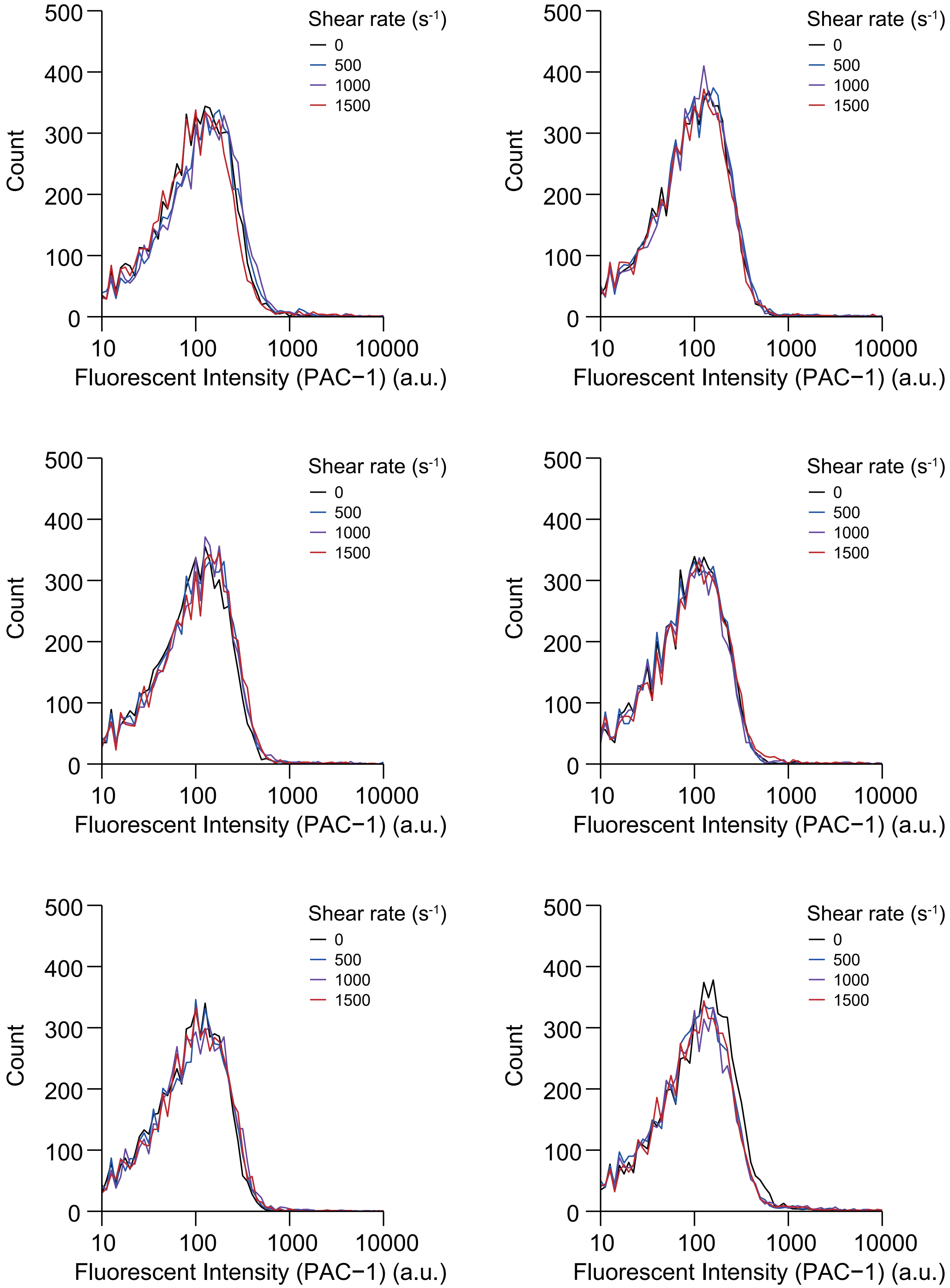
Shear rate (s-1)
0
500
1000
1500
Count
0
100
200
300
400
500
10
100
1000
10000
Fluorescent Intensity (PAC−1) (a.u.)

Supplementary Figure S5 | Fluorescence histograms of PAC-1 under shear for individual donors.

Fluorescence histograms from all six donors (10,000 events per condition) for PAC-1 at 0, 500, 1000, and 1500 s^{-1}. The activation threshold for PAC-1 is shown in Supplementary Figure S4(a).

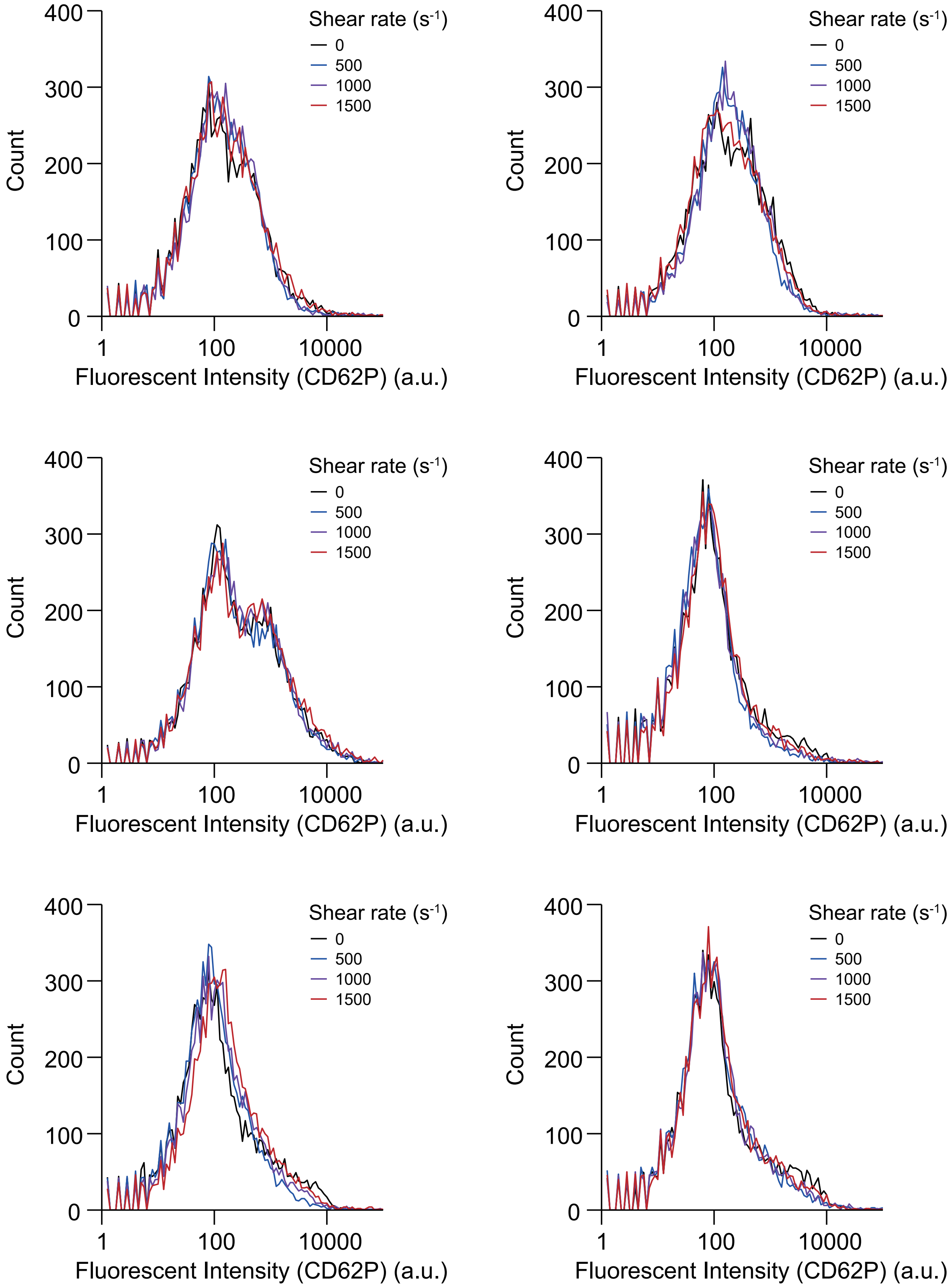
Shear rate (s-1)
0
500
1000
1500
Count
Fluorescent Intensity (CD62P) (a.u.)
0
100
200
300
400
1
100
10000
Shear rate (s-1)
0
500
1000
1500
Count
Fluorescent Intensity (CD62P) (a.u.)
0
100
200
300
400
1
100
10000
Shear rate (s-1)
0
500
1000
1500
Count
Fluorescent Intensity (CD62P) (a.u.)
0
100
200
300
400
1
100
10000
Shear rate (s-1)
0
500
1000
1500
Count
Fluorescent Intensity (CD62P) (a.u.)
0
100
200
300
400
1
100
10000
Shear rate (s-1)
0
500
1000
1500
Count
Fluorescent Intensity (CD62P) (a.u.)
0
100
200
300
400
1
100
10000
Shear rate (s-1)
0
500
1000
1500
Count
Fluorescent Intensity (CD62P) (a.u.)
0
100
200
300
400
1
100
10000

Supplementary Figure S6 | Fluorescence histograms of CD62P under shear for individual donors.

Fluorescence histograms from all six donors (10,000 events per condition) for CD62P at 0, 500, 1000, and 1500 s^{-1}. The activation threshold for CD62P is shown in Supplementary Figure S4(b).

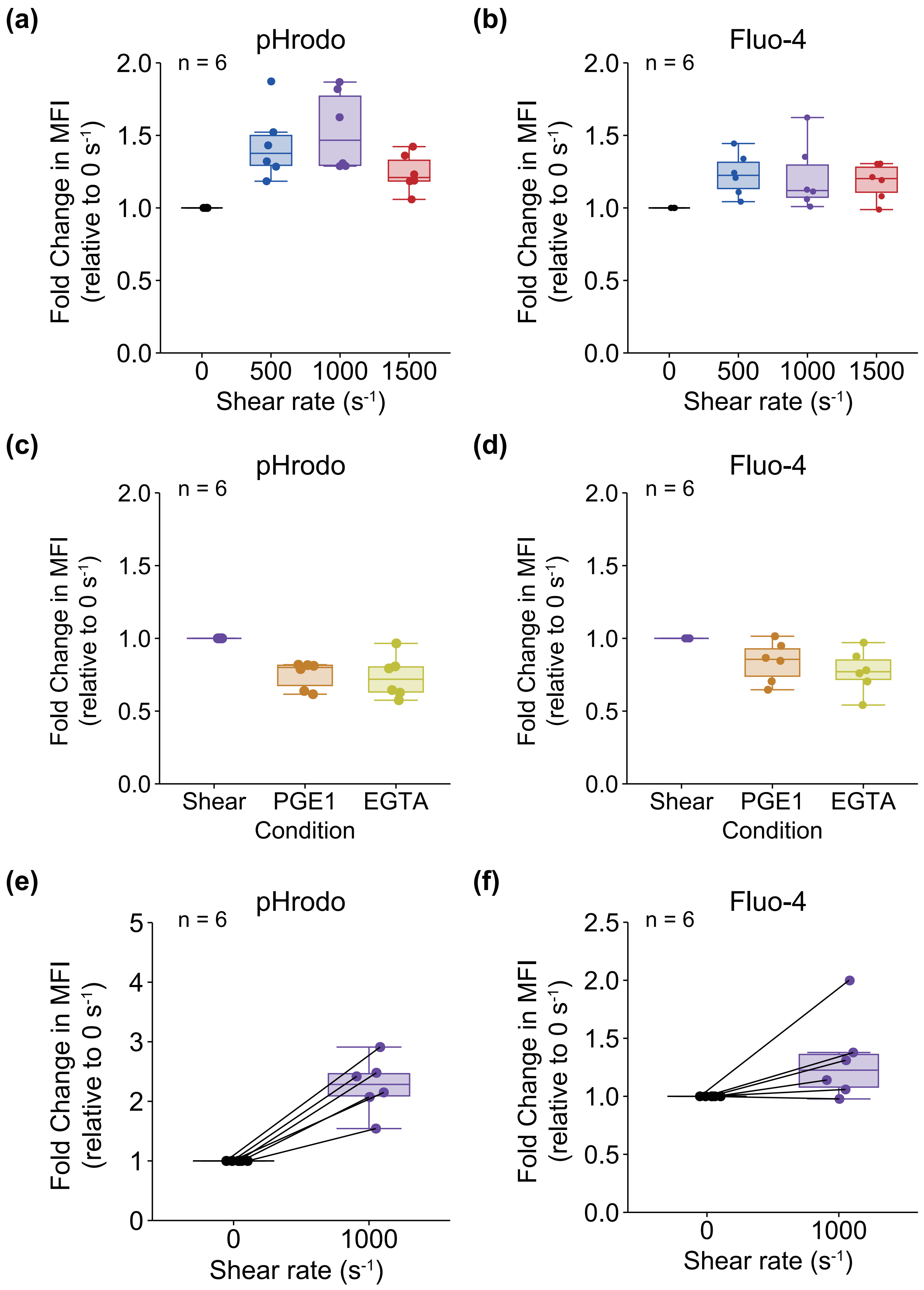

(a)
pHrodo
n = 6
Fold Change in MFI (relative to 0 s-1)
Shear rate (s-1)
0
500
1000
1500
(b)
Fluo-4
n = 6
Fold Change in MFI (relative to 0 s-1)
Shear rate (s-1)
(c)
pHrodo
n = 6
Shear
PGE1
EGTA
Condition
(d)
Fluo-4
n = 6
Shear
PGE1
EGTA
Condition
(e)
pHrodo
n = 6
Shear rate (s-1)
(f)
Fluo-4
n = 6
Shear rate (s-1)

Supplementary Figure S7 | Re-analysis of data using the 0 s⁻¹ condition as the reference. The mean fluorescence intensity (MFI) of (a) pHrodo uptake and (b) Fluo-4-based calcium response in human platelets (10,000 events) exposed to 0, 500, 1000, and 1500 s^{-1} shear. (c) pHrodo-dextran uptake and (d) Fluo-4-based calcium measurements under 1000 s^{-1} shear in the presence of PGE1 or EGTA. (e) pHrodo-dextran uptake and (f) Fluo-4 signals (MFI) in washed platelets resuspended in HEPES buffer and exposed to 0 or 1000 s^{-1} shear. All data are expressed as fold change relative to the 0 s^{-1} condition (*n* = 6 donors). A re-analysis using this reference did not alter the overall trends or distributions compared with those obtained using the external baseline.

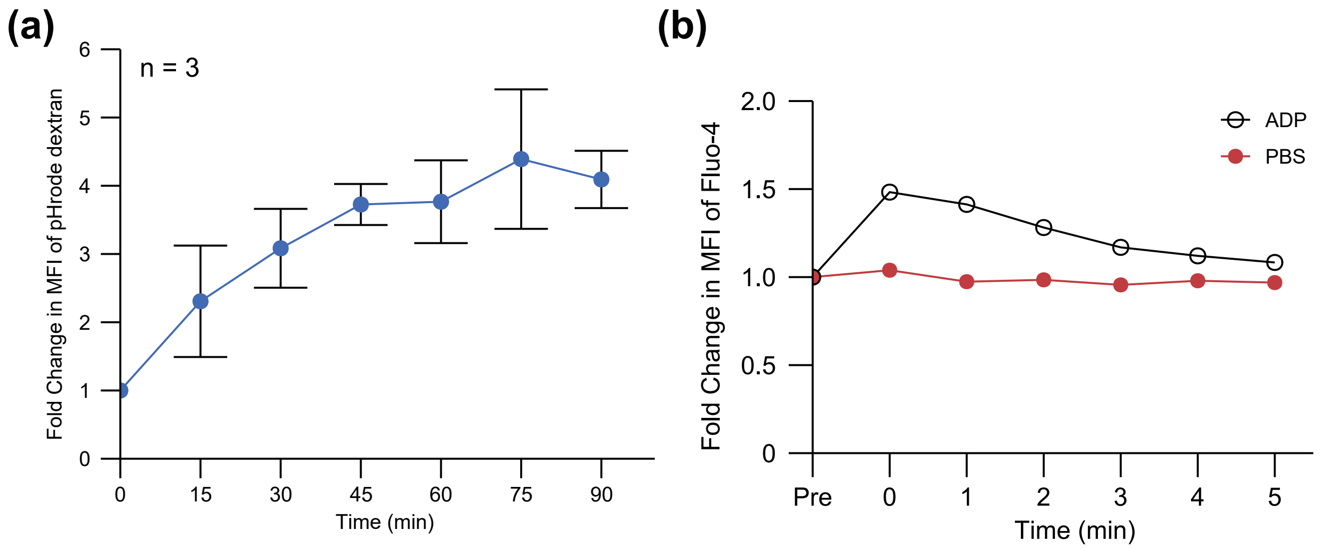
(a)
n = 3
Fold Change in MFI of pHrode dextran
Time (min)
0
1
2
3
4
5
6
15
30
45
60
75
90
(b)
Fold Change in MFI of Fluo-4
Time (min)
ADP
PBS
2.0
1.5
1.0
0.5
Pre

Supplementary Figure S8 | Time-dependent shear-free uptake and ADP-induced Ca^{2+} elevation.

(a) Time course (0–90 min) of pHrodo uptake under no-shear conditions (representative n = 3), shown as mean ± SD. (b) Representative Fluo-4 response to ADP stimulation (n = 1). These data characterize baseline pinocytosis behavior and validate Fluo-4 as an indicator of intracellular calcium dynamics.

Supplementary Table 1. Statistical analysis of shear rate–dependent platelet pinocytosis and intracellular Ca^{2+} elevation.

Figure	Comparison	Test	p value	Effect size	95% CI (Bootstrap, 5000 interactions)
Figure 1(a)	—	Friedman	**0.004	Kendall's W = 0.732	—
	500 s^{-1} vs 0 s^{-1}	Dunn + Bonferroni	*0.024	Rank-biserial r = 0.899	[1.00, 1.00]
	1000 s^{-1} vs 0 s^{-1}	Dunn + Bonferroni	*0.024	Rank-biserial r = 0.899	[1.00, 1.00]
	1500 s^{-1} vs 0 s^{-1}	Dunn + Bonferroni	0.754	Rank-biserial r = 0.899	[1.00, 1.00]
	1000 s^{-1} vs 500 s^{-1}	Dunn + Bonferroni	> 0.99	Rank-biserial r = 0.300	[-0.52, 1.00]
	1500 s^{-1} vs 500 s^{-1}	Dunn + Bonferroni	> 0.99	Rank-biserial r = -0.689	[-1.00, -0.20]
	1500 s^{-1} vs 1000 s^{-1}	Dunn + Bonferroni	0.235	Rank-biserial r = -0.813	[-1.00, -0.43]
Figure 1(b)	—	Friedman	*0.014	Kendall's W = 0.589	—
	500 s^{-1} vs 0 s^{-1}	Dunn + Bonferroni	0.096	Rank-biserial r = 0.899	[1.00, 1.00]
	1000 s^{-1} vs 0 s^{-1}	Dunn + Bonferroni	0.224	Rank-biserial r = 0.899	[1.00, 1.00]
	1500 s^{-1} vs 0 s^{-1}	Dunn + Bonferroni	0.247	Rank-biserial r = 0.813	[0.43, 1.00]
	1000 s^{-1} vs 500 s^{-1}	Dunn + Bonferroni	> 0.99	Rank-biserial r = 0.128	[-0.90, 0.71]
	1500 s^{-1} vs 500 s^{-1}	Dunn + Bonferroni	> 0.99	Rank-biserial r = -0.556	[-1.00, 0.14]
	1500 s^{-1} vs 1000 s^{-1}	Dunn + Bonferroni	> 0.99	Rank-biserial r = -0.214	[-1.00, 0.71]

Supplementary Table 2. Effects of calcium suppression on shear-induced platelet pinocytosis and Ca^{2+} signaling.

Figure	Comparison	Test	p value	Effect size	95% CI (Bootstrap, 5000 interactions)
Figure 2(a)	—	Friedman	*0.011	Kendall's W = 0.750	—
	PGE1 vs Shear	Dunn + Bonferroni	*0.018	Rank-biserial r = -0.899	[-1.00, -1.00]
	EGTA vs Shear	Dunn + Bonferroni	*0.018	Rank-biserial r = -0.899	[-1.00, -1.00]
	EGTA vs PGE1	Dunn + Bonferroni	> 0.99	Rank-biserial r = -0.128	[- 0.71, 0.91]
Figure 2(b)	—	Friedman	**0.006	Kendall's W = 0.861	—
	PGE1 vs Shear	Dunn + Bonferroni	0.223	Rank-biserial r = -0.813	[-1.00, -0.43]
	EGTA vs Shear	Dunn + Bonferroni	*0.028	Rank-biserial r = -0.899	[-1.00, -1.00]
	EGTA vs PGE1	Dunn + Bonferroni	> 0.99	Rank-biserial r = -0.899	[-1.00, -1.00]

Supplementary Table 3. Analysis of platelet activation markers under shear exposure.

Figure	Comparison	Test	p value	Effect size	95% CI (Bootstrap, 5000 interactions)
Figure 3(a)	—	Friedman	0.572	Kendall's W = 0.111	—
	500 s^{-1} vs 0 s^{-1}	Dunn + Bonferroni	> 0.99	Rank-biserial r = 0.300	[-0.52, 1.00]
	1000 s^{-1} vs 0 s^{-1}	Dunn + Bonferroni	> 0.99	Rank-biserial r = 0.471	[-0.43, 1.00]
	1500 s^{-1} vs 0 s^{-1}	Dunn + Bonferroni	> 0.99	Rank-biserial r = 0.214	[-0.62, 1.00]
	1000 s^{-1} vs 500 s^{-1}	Dunn + Bonferroni	> 0.99	Rank-biserial r = 0.471	[-0.43, 1.00]
	1500 s^{-1} vs 500 s^{-1}	Dunn + Bonferroni	> 0.99	Rank-biserial r = 0.043	[-0.71, 1.00]
	1500 s^{-1} vs 1000 s^{-1}	Dunn + Bonferroni	> 0.99	Rank-biserial r = 0.214	[-1.00, 0.71]
Figure 3(b)	—	Friedman	**0.002	Kendall's W = 0.811	—
	500 s^{-1} vs 0 s^{-1}	Dunn + Bonferroni	> 0.99	Rank-biserial r = 0.899	[-1.00, -1.00]
	1000 s^{-1} vs 0 s^{-1}	Dunn + Bonferroni	> 0.99	Rank-biserial r = 0.728	[-1.00, -0.14]
	1500 s^{-1} vs 0 s^{-1}	Dunn + Bonferroni	> 0.99	Rank-biserial r = 0.043	[-0.90, 0.81]
	1000 s^{-1} vs 500 s^{-1}	Dunn + Bonferroni	> 0.99	Rank-biserial r = 0.899	[-0.90, 0.81]
	1500 s^{-1} vs 500 s^{-1}	Dunn + Bonferroni	> 0.99	Rank-biserial r = 0.899	[1.00, 1.00]
	1500 s^{-1} vs 1000 s^{-1}	Dunn + Bonferroni	> 0.99	Rank-biserial r = 0.899	[1.00, 1.00]

Supplementary Table 4. Effects of plasma removal on platelet pinocytosis and intracellular Ca^{2+} levels.

Figure	Comparison	Test	p value	Effect size	95% CI (Bootstrap, 5000 interactions)
Figure 4(a)	1000 s^{-1} vs 0 s^{-1}	Wilcoxon signed-rank test	*0.036	Rank-biserial r = 0.899	[1.00, 1.00]
Figure 4(b)	1000 s^{-1} vs 0 s^{-1}	Wilcoxon signed-rank test	0.059	Rank-biserial r = 0.813	[0.43, 1.00]
